# CpG 684: an effective adjuvant for the inactivated COVID-19 vaccine in mice

**DOI:** 10.2217/fvl-2022-0172

**Published:** 2023-06-01

**Authors:** Jiandong Liu, Tianle Cang, Congli Jiang, Kelei Li, Siyuan Liu, Haixin Wang, Meirong Wang, Yan Chen, Yan Shao, Jiankai Liu

**Affiliations:** 1 Beijing Minhai Biotechnology Co. Ltd, Beijing, 102600, China; 2 Shenzhen Kangtai Biological Products Co., Ltd, Guangzhou, Shenzhen, 518000, China; 3 Jiangsu Taipuriu Biotechnology Co., Ltd, Taizhou Pharmaceutical City, Jiangsu, 225300, China

**Keywords:** CpG, neutralizing antibody, SARS-COV-2, vaccine, variants

## Abstract

**Aim:**

This study used CpG 684 as adjuvant of inactivated COVID-19 vaccine to detect a humoral and cellular immune response in mice.

**Materials & methods:**

We used 10 and 20 µg CpG 684 as adjuvants of an inactivated COVID-19 vaccine to immunize mice. IgG, IgG1, IgG2a, IgG2b and IgM binding antibodies were detected in serum by ELISA. The IFN-γ cytokine was detected by ELISPOT.

**Results:**

CpG 684 improved spike-specific IgG and IgM subtype binding antibodies and increased the neutralizing antibody titer against prototype, Delta and Beta strains. CpG 684 also improved cellular immune response.

**Conclusion:**

CpG 684 is an effective adjuvant for inactivated COVID-19 vaccine.

COVID-19 has been a serious health threat to people all over the world. Cases of infection by SARS-COV-2 have exceeded 500 million and there have been more than 6 million deaths caused by COVID-19 [1]. Throughout the COVID-19 pandemic, new variants appeared frequently and they continue to do so [2–4]. An effective COVID-19 vaccine is essential, and so far, multiple different types of vaccines have been licensed including mRNA, virus vector, recombinant protein and inactivated vaccines [5–10]. The protection rate of different vaccines vary, and mutations of the virus strain can reduce the effectiveness of vaccines [11–13]. To overcome this, it is useful to improve the humoral and cellular immune response induced by a vaccine.

CpG-containing synthetic phosphoguanine oligodeoxynucleotides (CpG ODNs) are comprised of cytosine and guanine-enriched unmethylated ssDNA sequences [14]. As CpG is the pathogen-associated molecular pattern of the toll-like receptor 9 [15], previous studies in other vaccines have demonstrated that CpG can enhance the humoral and cellular immune response to vaccines. CpG ODNs improve the activation and inhibits apoptosis of B cells, enhances the IgG subclass switch and induces the maturation and differentiation of dendritic cells [16–20]. The CpG 1018 adjuvant hepatitis B vaccine manufactured by Dynavax has been approved by US FDA for marketing in 2017 [21], and CpG 684 has proved its adjuvant effect in hepatitis B vaccines [22]. To increase the immune response to the inactivated COVID-19 vaccine, this study investigates the potential for CpG 684 as an adjuvant.

## Materials & methods

### Materials

Specific pathogen-free female Balb/c mice were purchased from Charles River Laboratories in China and maintained by the animal center of Beijing Minhai Biotechnology Co., Ltd (Beijing, China) inactivated COVID-19 vaccines using the prototype virus strain were manufactured by Shenzhen Kangtai Biological Products Co., Ltd (Shenzhen, China). CpG 684 was manufactured by Jiangsu Taipurui Biotechology Co., Ltd (Jiangyin, China).

### Vaccination

Specific pathogen-free Balb/c mice were separated into groups of 10, and each group was separately immunized by the intramuscular route with: normal saline; the inactivated COVID-19 vaccine; the inactivated COVID-19 vaccine and CpG 10 µg; and the inactivated COVID-19 vaccine and CpG 20 µg. The immunizing dose of inactivated COVID-19 vaccine contained 1 µg vaccine antigen and 50 µg aluminum hydroxide. Mice were immunized twice with a 28-day interval. Serum and spleen cells were obtained two weeks after the last immunization.

All animal experiments were conducted in accordance with relevant guidelines and regulations.

### ELISA assay for detecting SARS-COV-2 spike-specific binding antibody

SARS-COV-2 wildtype strain spike protein (Sino Biological Inc., China) was coated in 0.1 M sodium bicarbonate buffer solution (pH 9.6). The final concentration of spike protein was 1 μg/ml. About 100 μl was seeded into each well of a 96-well high-binding ELISA plate (Costar; NY, USA). The plates were incubated overnight at 4°C. After incubation, plates were washed three-times with PBST (phosphate-buffered saline [PBS] containing 0.05% Tween 20). The plates were locked with blocking solution (PBS containing 2% bovine serum albumin) and incubated for 1 h at 37°C. Serum was diluted 1:1000 with blocking solution and 100 μl of diluted serum was added to each well, followed by incubation at 37°C for 1 h. After incubation, the plates were washed five-times with PBST, and 100 μl of peroxidase-conjugated antimouse immunoglobulin G, G1, G2a, G2b, G3, A and M antibody were diluted 1:5,000 with blocking solution. respectively. About 100 μl antibody dilution solution was added to the plates and these were incubated at 37°C for 1 h. The plates were then washed five-times with PBST solution and 100 μl of 3,3′,5,5′-tetramethylbenzidine substrate was added to each well. The plates were incubated at room temperature for 5 min. The color reaction was terminated with 50 μl 2 mol/l sulfuric acid. The optical density at 450 nm was detected with an automated plate reader (Biotek Agilent, VT, USA).

### Neutralizing antibody assay

SARS-COV-2 prototype, Delta and Beta strain live viruses were used to detect neutralizing antibody titer from serum. Prior to the experiment, the serum was inactivated for 30 min at 56°C. First, the serum samples of mice were diluted in the first two columns of a 96-well plate at 1:4 dilution with cell culture maintenance medium. To each remaining well, 200 ul solution was added and the sample was diluted by twofold serial dilutions. Each well contained equal volumes of viral solution, for a final concentration of 100 TCID50/well. The plates were then incubated at 37°C for 1 h. Vero cells were seeded in 96-well plates and reached about 80% confluence before infection. The incubated virus-serum mixtures were added to 96-well cell plates and incubated at 37°C for 4 days. The cytopathic effect of each well was observed under a microscope and the neutralizing antibody titer against SARS-COV-2 was recorded as the highest dilution of serum that showed 50% inhibition activity against SARS-CoV-2.

### ELISPOT assay for detecting IFN-γ

A commercial kit (Mouse IFN-γ ELISPOT Pair, BD, USA) was used to detect IFN-γ secreted by splenic immune cells. The antibody to IFN-γ was then diluted at 1:200 dilution with PBS, 100 μl of diluted antibody solution was added to each well of an ELISPOT plate and the plate was incubated overnight at 4°C. After incubation, the coating solution was discarded and the plate was washed once with 200 ul/well blocking solution (1640 solution containing 10% fetal bovine serum [FBS] and 1% L-glutamine). About 200 μl of blocking solution was then added to each well, and the plate was incubated for 2 h at room temperature. Splenic cells of vaccine-immunized mice were thawed with 1640 medium (1640 solution containing 10% FBS and 1% L-glutamine) and the cells were counted by a cell counter. Cell suspensions were prepared at 10^5^ cells/ml density. About 100 ul of each cell suspension were then added to the wells of the ELISPOT plate. Each splenic cells suspension was added to three wells, one as a negative control and two as experiment samples. The experiment sample wells were stimulated by 20 µg SARS-COV-2 spike antigen peptide. The ELISPOT plate was then incubated at 37°C and 5% CO_2_ for 20 h. Cell suspension was the discarded and the wells were washed twice with 200 ul deionized water. The plate was then washed three-times with 200 ul PBST solution. The biotinylated antimouse IFN-γ antibody was diluted with dilution buffer (PBS containing 10% FBS) at a ratio of 1:250. About 100 ul diluted antibody solution was added to each well and the plate was incubated at room temperature for 2 h. The plate was washed three-times with 200 ul PBST solution. The streptavidin-HRP antibody was diluted with dilution buffer (PBS containing 10% FBS) at a ratio of 1:100. 100 ul diluted antibody solution was added to each well and the plate was incubated at room temperature for 1 h. Streptavidin-HRP antibody solution was discarded and the plate was washed six-times with 200 ul PBST solution, 100 ul AEC substrate solution was added and spots were allowed to develop for 30 min in the dark. The substrate color reaction was stopped by washing the wells with deionized water. The plate was air-dried overnight at room temperature in the dark. The spots were counted by ELISPOT reader (AID, Germany).

### Statistical analysis

Spike protein-specific binding antibody data were presented as mean ± standard deviation. The neutralizing antibody data were presented as geometric mean. The differences between the control group and the experimental groups were analyzed using an unpaired *t*-test. Data were considered to be statistically significant at p < 0.05.

## Results

### Spike-specific IgG & IgG binding antibody response

**Figure 1.**
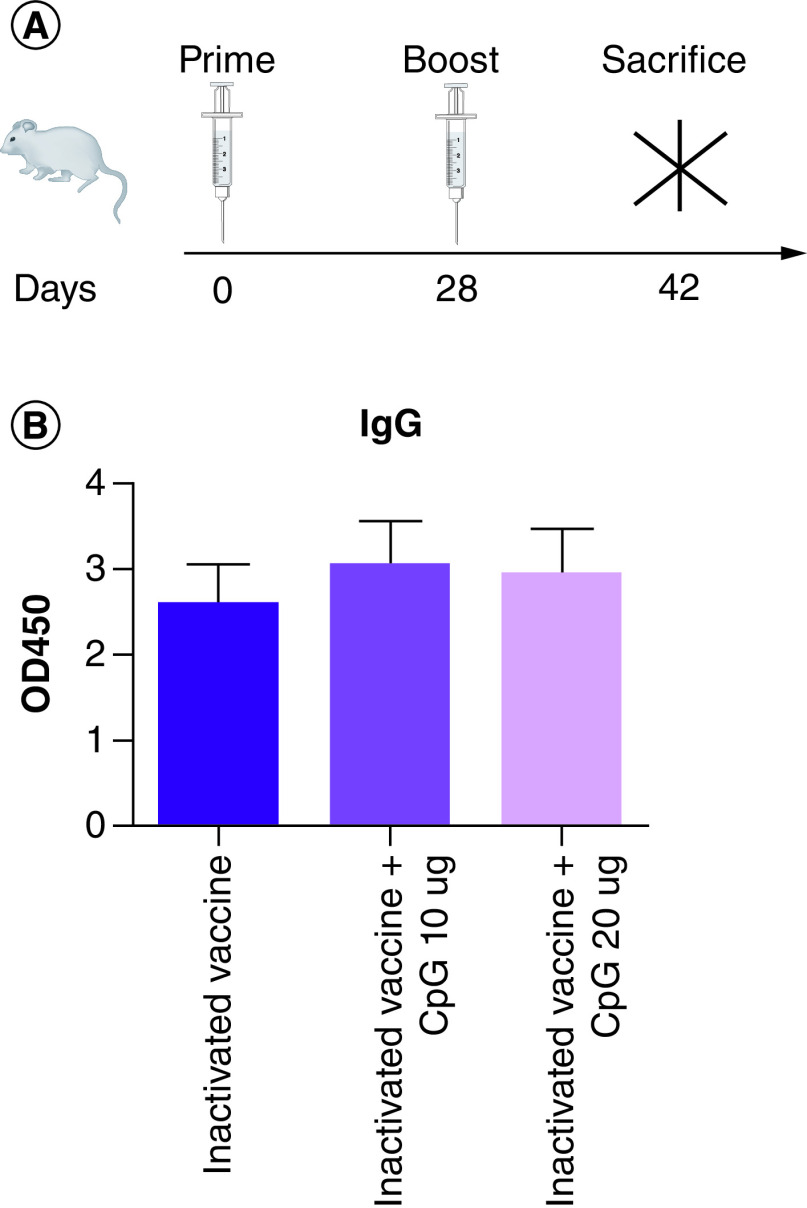
Mouse immunity and gE protein-specific IgG binding antibodies. **(A)** Immunization experiment. **(B)** Spike-specific IgG binding antibody responses.

**Figure 2.**
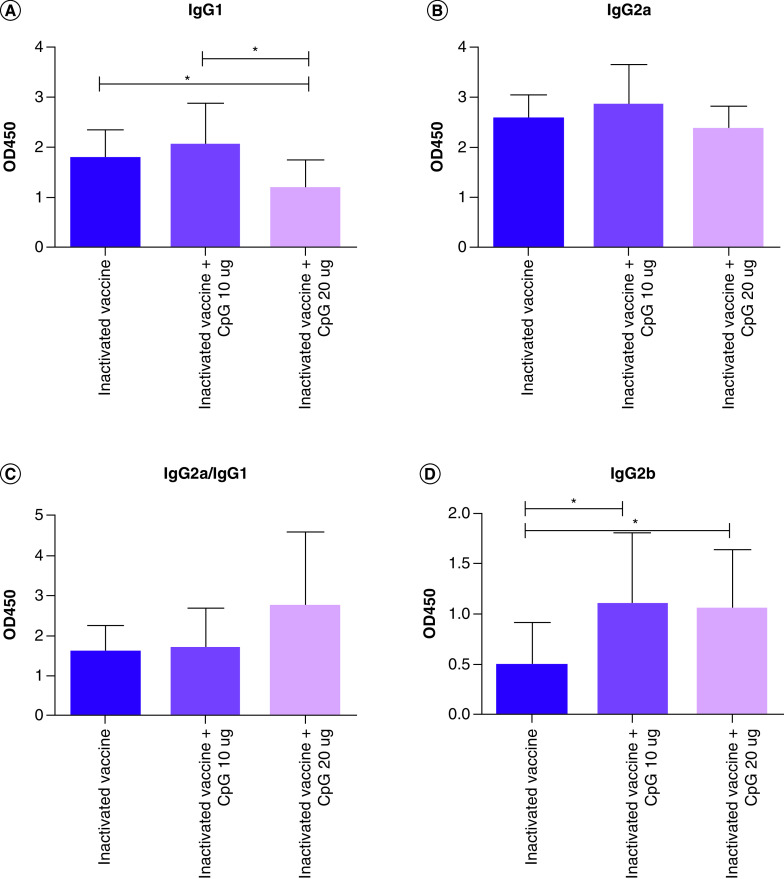
Spike-specific IgG subclass binding antibody responses. **(A)** IgG1 binding antibody responses. **(B)** IgG2a binding antibody responses. **(C)** IgG2a/IgG1 ratio. **(D)** IgG2b binding antibody responses. *p < 0.05.

After two doses of the vaccine, mice serum was tested for spike-specific IgG and IgG binding antibody response (Figure 1A). The results showed that CpG increased the IgG binding antibody response to the inactivated COVID-19 vaccine (Figure 1B). The IgG1 binding antibody response to inactivated COVID-19 vaccine and CpG 20 µg was significantly lower than to the inactivated COVID-19 vaccine and the inactivated COVID-19 vaccine and CpG 10 µg group (Figure 2A). There were no differences in the IgG2a binding antibody response among the three groups (Figure 2B). The IgG2a/IgG1 ratio in the inactivated COVID-19 vaccine and CpG 20 µg group was higher than in the inactivated COVID-19 vaccine and inactivated COVID-19 vaccine and CpG 10 µg group (Figure 2C). The data indicates that CpG changed the antibody subtype ratio in response to the inactivated COVID-19 vaccine and increased functional antibody ratio. The IgG2b binding antibody response to the inactivated COVID-19 vaccine was significantly lower than to the inactivated COVID-19 vaccine and CpG 20 µg and the inactivated COVID-19 vaccine and CpG 10 µg (Figure 2D).

### Spike-specific IgM binding antibody response

**Figure 3.**
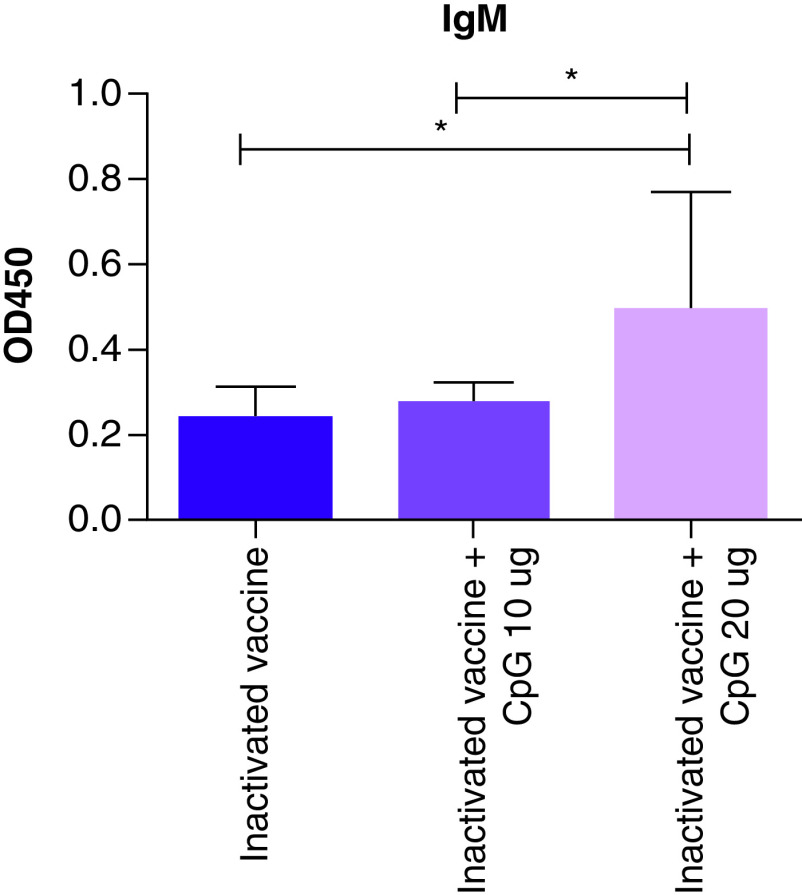
Spike-specific IgM binding antibody responses. *p < 0.05.

There were significant differences in the IgM binding antibody response to inactivated COVID-19 vaccine and CpG 10 µg, the inactivated COVID-19 vaccine and CpG 20 µg and the inactivated COVID-19 vaccine (Figure 3). The IgM binding antibody response to the inactivated COVID-19 vaccine was significantly lower than the inactivated COVID-19 vaccine and CpG 10 µg and inactivated COVID-19 vaccine and CpG 20 µg.

### Neutralizing antibody response

**Figure 4.**
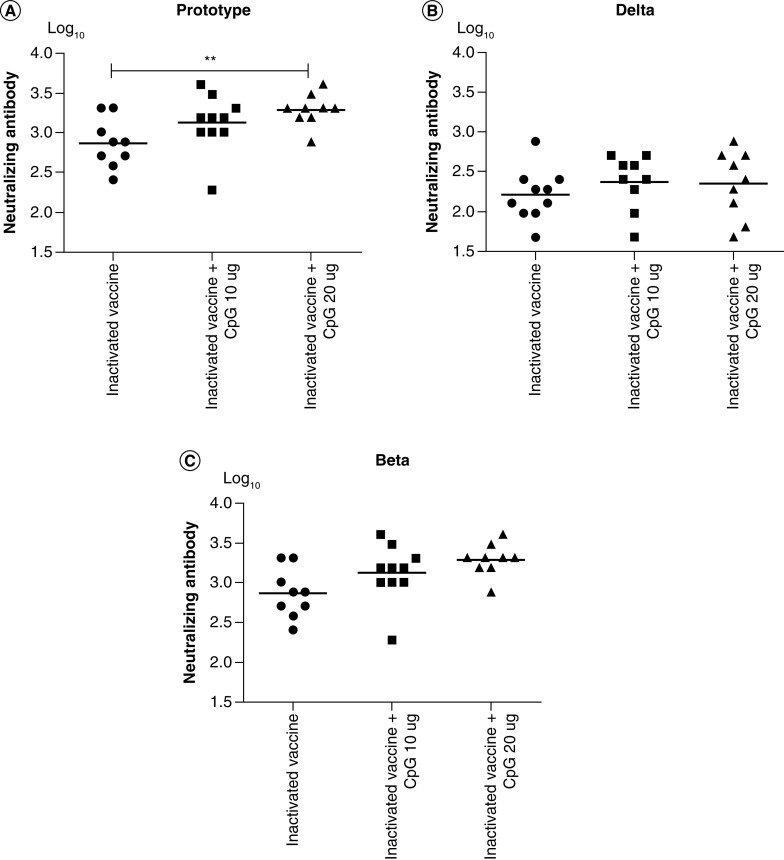
The neutralizing antibody titer of mice sera against prototype, Delta and Beta strains of SARS-COV-2. **(A)** The neutralizing antibody titer against prototype strain. **(B)** The neutralizing antibody titer against the Delta strain. **(C)** The neutralizing antibody titer against the Beta strain. **p < 0.01.

Neutralizing antibody titers against the prototype, Delta and Beta strains of SARS-COV-2 were detected by live virus cytopathic effect. The data showed that CpG increased the neutralizing antibody titer in all tested strains, with a significant difference in neutralizing antibodies against the prototype strain between the inactivated COVID-19 vaccine and the inactivated COVID-19 vaccine and CpG 20 µg (Figure 4A). The neutralizing antibody titer against the prototype strain in response to the inactivated COVID-19 vaccine and CpG 20 µg was 2.6-times higher than that to the inactivated COVID-19 vaccine (Table 1). The neutralizing antibody titer of CpG adjuvant-inactivated vaccines against Delta and Beta strains was higher than in the inactivated vaccine group. However, compared with the neutralizing antibody titer against the prototype strain, the decrease rate of neutralizing antibody titers against Delta and Beta strains in the CpG adjuvant-inactivated vaccine group was higher than that in the inactivated vaccine group (Table 1).

**Table 1.** Neutralizing antibody titer against SARS-COV-2.

Group	Virus type	Neutralizing antibody										GMT	Decrease rate
Inactivated vaccine	Prototype	256	768	1024	512	2048	512	2048	768	384		738	–
	Delta	96	96	192	192	48	256	256	128	768	128	163	4.52
	Beta	64	192	384	512	192	192	256	256	768	96	231	3.20
Inactivated vaccine+CpG 10 ug	Prototype	3062	1536	2048	1536	4096	1536	1536	1024	4096		2035	–
	Delta	256	256	512	384	384	192	48	96	512		236	8.64
	Beta	96	384	384	384	256	256	48	192	192		205	9.94
Inactivated vaccine+CpG 20 ug	Prototype	3062	4096	6144	768	6144	1024	1536	3062	3062		2596	–
	Delta	768	512	512	192	384	48	128	256	64		225	11.53
	Beta	768	192	768	768	512	192	128	768	768		442	5.87

GMT: Geometric mean titer.

### Cellular response

**Figure 5.**
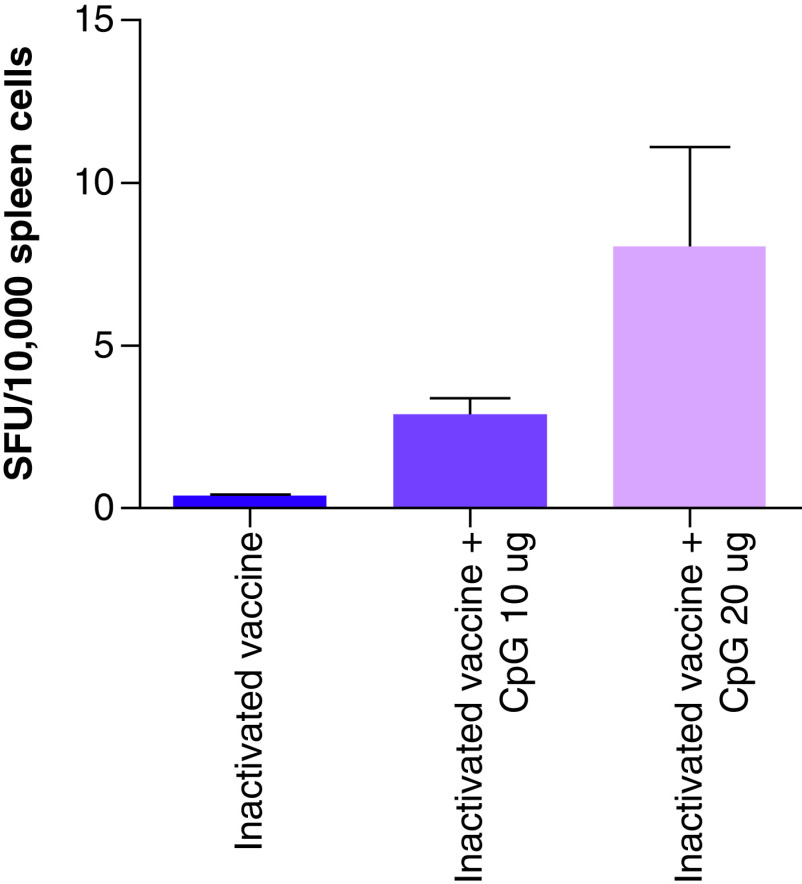
ELISPOT spots forming unit of IFN-γ. *p < 0.05; ***p < 0.001.

An ELISPOT assay was conducted to detect IFN-γ secreted by SARS-COV-2 vaccine immune splenic cells, and the data indicated that splenic cells of mice injected with the inactivated vaccine hardly secreted IFN-γ cytokines (Figure 5). CpG significantly increased IFN-γ spots, and the difference between the inactivated vaccine and both the inactivated vaccine and CpG 10 µg and the inactivated vaccine and CpG 20 µg was significant.

## Discussion

An effective vaccine is the best way to control the transmission of SARS-COV-2, yet the protective effect of the inactivated vaccine could be improved. CpG has been proven to improve humoral and cellular immune response in vaccine research by inducing the innate immune response to improve antigen presentation [23,24]. This study investigated the CpG adjuvant effect for the inactivated COVID-19 vaccine and found that CpG 684 can increase the neutralizing antibody and IgG2b and IgM binding antibody responses.

The current opinion is that neutralizing antibodies correlate to protection rate of the SARS-COV-2 vaccine [5–10]. In this study, we found that CpG 684 increased the spike-specific IgG2b and IgM binding and neutralizing antibody response. A recent study of COVID-19 vaccines showed that IgM, IgA and IgG antibodies may play an important role in the protective antiviral response of the body [25]. This change of the IgG2a/IgG1 ratio may contribute to an increase in neutralizing antibodies, as IgG2a/IgG1 ratio is an important indicator of T helper cell (Th) polarization: Th1 mediates the cellular immune response and mainly secretes IFN-γ, IL-2, TNF, α-IFN and stimulates B cells to produce IgG2a antibodies, whereas Th2 mediates the humoral immune response and mainly secrete IL-4, IL-5, IL-6, IL-10 and stimulate B cells to produce IgG1 antibodies [26–29]. Many studies have shown that changes in the immune response from Th1 to Th2 improve vaccine protection [30–32]. The shift in IgG2a/IgG1 ratio induced by the CpG adjuvant may result in the increase of neutralizing antibody titer observed in our study and may improve vaccine protection. It has been reported in other vaccine research that CpG can alter vaccine-induced IgG2a/IgG1 antibody ratio.

In terms of neutralizing antibodies, CpG adjuvants can enhance the neutralizing antibody response induced by the inactivated vaccine. The ratio between antigen and CpG has been correlated to neutralizing antibody titers [33], so optimizing the ratio of antigen and adjuvant may improve the neutralizing antibody titer of inactivated vaccines. The decrease rate of neutralizing antibodies against Delta and Beta variants in the CpG adjuvant-inactivated vaccine group was higher than that of the prototype strain, which may be because the antigen-presenting cells activated by CpG preferentially presented the dominant epitope of the prototypic strain.

The cellular immune response plays an important role in the immune protection of vaccines. An IFN-γ ELISPOT test was used to detect the cellular immune response to the CpG adjuvant and inactivated vaccine and indicated that the inactivated vaccine resulted in minimal secretion of IFN-γ cytokines. Although CpG 684 increased IFN-γ, the spot forming unit of IFN-γ induced by the CpG adjuvant-inactivated vaccine was still low. There may be two reasons for this. The first was that CpG 684 belongs to a B-type CpG, which trigger pDCs to differentiate and produce TNF-α and B cells to proliferate and secrete IgM. B-type CpGs are weak in inducing a cellular immune response. The second reason was that the number of splenic cells used in the ELISPOT test was 10^4^ cells per well, and the number of splenic cells routinely used in the ELISPOT test is 10^5^–10^6^ cells/ per well. Because the splenic cells underwent cryopreservation, the number of living cells decreased.

## Conclusion

In summary, the unmethylated CpG motif CpG 684, the PAMP of toll-like receptor-9, in this study was shown to improve the humoral and cellular immune response of inactivated COVID-19 vaccine. It may therefore be an effective adjuvant of the inactivated COVID-19 vaccine.

**Summary points**

- The data showed that CpG 684 can enhance IgG, IgG2b and IgM binding antibodies in response to the inactivated COVID-19 vaccine.
- The data showed that CpG 684 can change the ratio between IgG1 and IgG2a binding antibodies in response to the inactivated COVID-19 vaccine. The CpG 684 can alter immune response from Th2 to Th1.
- CpG 684 can enhance the neutralizing antibody titers of the inactivated COVID-19 vaccine against prototype, Delta and Beta strains.
- In summary, the CpG 684 is an effective adjuvant for the inactivated COVID-19 vaccine.

## References

[1] Mistry P, Barmania F, Mellet J SARS-CoV-2 variants, vaccines, and host immunity. Front. Immunol. 12, 809244 (2021). 35046961 10.3389/fimmu.2021.809244 PMC8761766

[2] Team CC-R. SARS-CoV-2 B.1.1.529 (Omicron) variant – United States, December 1–8, 2021. Morb. Mortal. Wkly Rep. 70, 1731–1734 (2021). 10.15585/mmwr.mm7050e1 PMC8675659 34914670

[3] Potdar V, Vipat V, Jadhav S Detection of SARS-CoV-2 variants in India from UK returnees. Infection 49, 1355–1359 (2021). 34160788 10.1007/s15010-021-01617-6 PMC8220361

[4] Adiga R, Nayak V. Emergence of novel SARS-CoV-2 variants in India: second wave. J. Infect. Dev. Ctries. 15, 1578–1583 (2021). 34898481 10.3855/jidc.15484

[5] Baden LR, El Sahly HM, Essink B Efficacy and safety of the mRNA-1273 SARS-CoV-2 vaccine. N. Engl. J. Med. 384, 403–416 (2021). 33378609 10.1056/NEJMoa2035389 PMC7787219

[6] Polack FP, Thomas SJ, Kitchin N Safety and Efficacy of the BNT162b2 mRNA COVID-19 vaccine. N. Engl. J. Med. 383, 2603–2615 (2020). 33301246 10.1056/NEJMoa2034577 PMC7745181

[7] Tanriover MD, Doganay HL, Akova M Efficacy and safety of an inactivated whole-virion SARS-CoV-2 vaccine (CoronaVac): interim results of a double-blind, randomised, placebo-controlled, Phase III trial in Turkey. Lancet 398, 213–222 (2020). 10.1016/S0140-6736(21)01429-X PMC8266301 34246358

[8] Logunov DY, Dolzhikova IV, Shcheblyakov DV Safety and efficacy of an rAd26 and rAd5 vector-based heterologous prime-boost COVID-19 vaccine: an interim analysis of a randomised controlled Phase III trial in Russia. Lancet 397, 671–681 (2021). 33545094 10.1016/S0140-6736(21)00234-8 PMC7852454

[9] Falsey AR, Sobieszczyk ME, Hirsch I Phase III safety and efficacy of AZD1222 (ChAdOx1 nCoV-19) COVID-19 vaccine. N. Engl. J. Med. 385, 2348–2360 (2021). 34587382 10.1056/NEJMoa2105290 PMC8522798

[10] Sadoff J, Gray G, Vandebosch A 2021 safety and efficacy of single-dose Ad26.COV2.S vaccine against COVID-19. N. Engl. J. Med. 384, 2187–2201 (2021). 33882225 10.1056/NEJMoa2101544 PMC8220996

[11] Bian L, Gao F, Zhang J Effects of SARS-CoV-2 variants on vaccine efficacy and response strategies. Expert Rev. Vaccines 20, 365–373 (2021). 33851875 10.1080/14760584.2021.1903879 PMC8054487

[12] Ikegame S, Siddiquey MNA, Hung CT Neutralizing activity of sputnik V vaccine sera against SARS-CoV-2 variants. Nat. Commun. 12, 4598 (2021). 34312390 10.1038/s41467-021-24909-9 PMC8313705

[13] Sharun K, Tiwari R, Dhama K Emerging SARS-CoV-2 variants: impact on vaccine efficacy and neutralizing antibodies. Hum. Vaccin. Immunother. 17, 3491–3494 (2021). 34161189 10.1080/21645515.2021.1923350 PMC8240541

[14] Bode C, Zhao G, Steinhagen F CpG DNA as a vaccine adjuvant. Expert Rev. Vaccines 10, 499–511 (2011). 21506647 10.1586/erv.10.174 PMC3108434

[15] King K, Larsen BB, Gryseels S Coevolutionary analysis implicates toll-like receptor 9 in papillomavirus restriction. mBio 13, e0005422 (2022). 35311536 10.1128/mbio.00054-22 PMC9040848

[16] Yi AK, Chang M, Peckham DW CpG oligodeoxyribonucleotides rescue mature spleen B cells from spontaneous apoptosis and promote cell cycle entry. J. Immunol. 160, 5898–5906 (1998). 9637502

[17] Davis HL, Weeratna R, Waldschmidt TJ 1998 CpG DNA is a potent enhancer of specific immunity in mice immunized with recombinant hepatitis B surface antigen. J. Immunol. 160, 870–876 (1998). 9551923

[18] Liu N, Ohnishi N, Ni L CpG directly induces T-bet expression and inhibits IgG1 and IgE switching in B cells. Nat. Immunol. 4, 687–693 (2003). 12766768 10.1038/ni941

[19] He B, Qiao X, Cerutti A. CpG DNA induces IgG class switch DNA recombination by activating human B cells through an innate pathway that requires TLR9 and cooperates with IL-10. J. Immunol. 173, 4479–4491 (2004). 15383579 10.4049/jimmunol.173.7.4479

[20] Hartmann G, Weiner GJ, Krieg AM. CpG DNA: a potent signal for growth, activation, and maturation of human dendritic cells. Proc. Natl Acad. Sci. USA 96, 9305–9310 (1999). 10430938 10.1073/pnas.96.16.9305 PMC17777

[21] FDA. Vaccines-blood-biologics/vaccines/heplisav-b. https://www.fda.gov/vaccines-blood-biologics/vaccines/heplisav-b

[22] Liu L, Shen L, Liu X 2012 A safety study of a B-class CpG ODN in Sprague-Dawley rats. J. Appl. Toxico. 32, 60–71 (2012). 10.1002/jat.1683 21538408

[23] Gursel M, Verthelyi D, Klinman DM. CpG oligodeoxynucleotides induce human monocytes to mature into functional dendritic cells. Eur. J. Immunol. 32, 2617–2622 (2002). 12207346 10.1002/1521-4141(200209)32:9<2617::AID-IMMU2617>3.0.CO;2-F

[24] Heger E, Schuetz A, Vasan S. HIV vaccine efficacy trials: RV144 and beyond. Adv. Exp. Med. Biol. 1075, 3–30 (2018). 30030787 10.1007/978-981-13-0484-2_1

[25] Azkur AK, Akdis M, Azkur D Immune response to SARS-CoV-2 and mechanisms of immunopathological changes in COVID-19. Allergy 75(7), 1564–1581 (2020). 32396996 10.1111/all.14364 PMC7272948

[26] Romagnani S. T-cell subsets (Th1 versus Th2). Ann. Allergy Asthma Immunol. 85, 9–18 (2000). 10923599 10.1016/S1081-1206(10)62426-X

[27] Romagnani S. Th1/Th2 cells. Inflamm. Bowel Dis. 5, 285–294 (1999). 10579123 10.1097/00054725-199911000-00009

[28] Infante-Duarte C, Kamradt T. Th1/Th2 balance in infection. Springer Semin. Immunopathol. 21, 317–338 (1999). 10666776 10.1007/BF00812260

[29] Kidd P. Th1/Th2 balance: the hypothesis, its limitations, and implications for health and disease. Altern. Med. Rev. 8, 223–246 (2003). 12946237

[30] Yang L, Lian Z, Zhang B Effect of ligustrazine nanoparticles on Th1/Th2 balance by TLR4/MyD88/NF-kappaB pathway in rats with postoperative peritoneal adhesion. BMC Surg. 21, 211 (2021). 33902534 10.1186/s12893-021-01201-7 PMC8077798

[31] Yamaguchi K, Hisano M, Isojima S Relationship of Th1/Th2 cell balance with the immune response to influenza vaccine during pregnancy. J. Med. Virol. 81, 1923–1928 (2000). 10.1002/jmv.21620 19774681

[32] Pacheco IL, Abril N, Morales-Prieto N Th1/Th2 balance in the liver and hepatic lymph nodes of vaccinated and unvaccinated sheep during acute stages of infection with Fasciola hepatica. Vet. Parasitol. 238, 61–65 (2017). 28385539 10.1016/j.vetpar.2017.03.022

[33] Kuo T-Y, Lin M-Y, Coffman RL Development of CpG-adjuvanted stable prefusion SARS-CoV-2 spike antigen as a subunit vaccine against COVID-19. Sci. Rep. 10(1), 20085 (2020). 33208827 10.1038/s41598-020-77077-z PMC7676267

